# EARLY MOBILIZATION IN PATIENTS WITH ANEURYSMAL SUBARACHNOID HAEMORRHAGE MAY IMPROVE FUNCTIONAL STATUS AND REDUCE CEREBRAL VASOSPASM RATE: A SYSTEMATIC REVIEW WITH META-ANALYSIS

**DOI:** 10.2340/jrm.v56.41225

**Published:** 2024-10-18

**Authors:** Adela FOUDHAILI, Brice LECLERE, Florence MARTINACHE, Anthony CHAUVIN, Damien VITIELLO, Benjamin G. CHOUSTERMAN

**Affiliations:** 1 Department of Physical Medicine and Rehabilitation, AP-HP, CHU Lariboisière, Paris, France; 2 Université Paris Cité, Inserm, MASCOT, Paris, France; 3 Université Paris Cité, Institut des Sciences du Sport-Santé de Paris, Paris, France; 4 Nantes Université, CHU Nantes, IICiMed, UR 1155, Nantes, France; 5 Université Paris-Saclay, CIAMS, Orsay, France; 6 Techno Concept, Manosque, France; 7 Department of Anesthesiology and Critical Care, AP-HP, CHU Bicêtre, Le-Kremlin-Bicêtre, France; 8 Department of Emergency, AP-HP, CHU Lariboisière, Paris, France; 9 Department of Anesthesiology and Critical Care, CHU Lariboisière, AP-HP, Paris, France

**Keywords:** critical care, early ambulation, physical therapy modalities, subarachnoid haemorrhage, systematic review, functional status, vasospasm

## Abstract

**Objective:**

The primary aim of this study was to evaluate the safety and efficacy of early mobilization in patients with aneurysmal subarachnoid haemorrhage.

**Design:**

Systematic review with meta-analysis of randomized controlled studies and observational studies.

**Patients:**

Patients with aneurysmal subarachnoid haemorrhage.

**Methods:**

PubMed, Embase, CINAHL, Web of Science, Pedro, and the Cochrane Library databases were searched. A systematic review and meta-analysis were performed. Screening and data extraction were performed by 2 independent reviewers.

**Results:**

Sixteen studies involving 1,757 patients were included. Meta-analysis of the data estimated that early mobilization improved mRS score at discharge (mean difference –1.39, 95% CI –2.51 to –0.28, I^2^ = 86%) and at 3 months (mean difference –1.10, 95% CI –1.54 to –0.66, I^2^ = 7%). Early mobilization was associated with a reduction in cerebral vasospasm rate, both radiological (OR 0.66, 95% CI 0.45 to 0.96, I^2^ = 7%) and clinical (OR 0.44, 95% CI 0.27 to 0.72, I^2^ = 8%); 6% of mobilization sessions involved adverse events, mostly haemodynamic changes.

**Conclusion:**

This review found moderate-quality evidence supporting the safety and effectiveness of early mobilization in patients with SAH. Further randomized controlled trials are needed to identify the appropriate mobilization strategy and confirm these results.

## LAY ABSTRACT

Early mobilization of patients admitted to the intensive care unit is now considered standard of care. However, for neurocritical patients, including those with subarachnoid haemorrhage (SAH), it remains unclear whether implementing early mobilization is safe and beneficial. This systematic review aimed to evaluate the safety and efficacy of early mobilization in this population. Several databases were screened to extract eligible articles, and 16 articles were included in this review. The results indicate that early mobilization in patients with SAH appears to be safe, not associated with major safety events, and improves the odds of functional independence. Standardization of the mobilization procedure seems essential to guarantee safety. However, the quality of evidence is moderate and warrants further studies to confirm these findings.

Subarachnoid haemorrhage (SAH) is a severe neurological condition, associated with high mortality and morbidity rates (1). While mortality has improved over the past decades, survivors still report significant disabilities and limitations in activities of daily living. About 20% of survivors are not fully independent 1 year after SAH, and 25% still have activity restrictions, such as difficulties returning to work (2, 3). After several years, patients with SAH still report limitations in participation in activities of daily living and work, and cognitive deficits. These patients also suffer from mood disorders, severe fatigue, and reduced quality of life (4–7).

The management of patients with SAH is complex, often requiring admission to intensive care units (ICUs) for close monitoring and treatment of potential complications. Over the past decades, the therapeutic approach to aneurysmal SAH has undergone major advancements. In contrast, the question of early mobilization of patients with SAH remains a relatively unexplored yet crucial area. The 2023 American Heart Association/American Stroke Association (AHA/ASA) recommendations on the management of patients with SAH advocate the implementation of an early mobilization protocol to improve functional outcome (8). However, these recommendations do not specify the mobilization modalities to be applied. It has been shown that the timing, intensity, and frequency of mobilization have a major impact on functional prognosis (9, 10). Identifying the optimal approach to mobilization is therefore essential.

In this context, this systematic review and meta-analysis aims to determine the impact of different early mobilization strategies of patients with SAH on (1) physical function and physical performance tasks; (2) cognitive function; (3) psychological state; (4) health-related quality of life; (5) adverse events related to mobilization; and (6) neurological complications.

## METHODS

This systematic review with meta-analysis was reported in accordance with the PRISMA statement (Preferred reporting Items for Systematic Reviews and Meta-analyses) and was pre-registered on PROSPERO (International prospective register of systematic reviews) with registration number CRD42022298976.

### Identification and selection of studies

The literature search was designed by the authors with assistance from a medical librarian. The search was executed in PubMed, Embase, CINAHL, Web of Science, Pedro, and the Cochrane Library databases in February 2022 and repeated prior to completion of analysis to identify studies that would have been published after the initial search. The last search was performed in February 2024. Snowballing and reverse snowballing were carried out to identify any other existing studies. The detailed search strategy can be found in the Supplementary Appendix.

### Criteria for considering studies

*Types of studies.* We searched for randomized controlled trials, non-randomized controlled trials, and observational studies written in English or French and published from 2001 to February 2024.

*Types of patients.* Adult patients with aneurysmal subarachnoid haemorrhage.

*Interventions.* Studies were selected for inclusion if they evaluated patients with aneurysmal subarachnoid haemorrhage who received early mobilization delivered by any members of the team. We considered interventions consisting of mobilization carried out earlier than those received by the control group, where applicable. Mobilization could be passive, assisted active, or active mobilizations, including but not restricted to passive/active exercises in bed, bed mobility training, dangling, passive/active transfers, passive/active exercises sitting out of bed, pre-gait exercises, ambulation. The comparator, where applicable, should have consisted of delayed mobilization or no mobilization.

We excluded case report studies, studies of mixed neurological populations, and studies that focused exclusively on head-of-bed changes.

*Types of outcome measures.* Eligible studies were those that reported at least 1 of the following endpoints: measures of physical function or physical performance tasks, cognitive function, psychological state, health-related quality of life or well-being, the occurrence of neurological complications, and adverse events related to mobilization.

Outcomes were categorized as follows:

- Physical function (defined as the ability to perform everyday activities) or physical performance tasks measured by a validated scale (e.g., Functional Independence Measure (FIM), Barthel Index, modified Rankin scale, walking tests, mobility scores) at discharge or during patient follow-up.
- Cognitive function measured by a validated scale (e.g., Montreal Cognitive Assessment [MoCA], Mini-Mental State Examination [MMSE]) at discharge or during patient follow-up.
- Psychological state measured by a validated scale (e.g., the Hospital Anxiety and Depression Scale [HADS], Impact of Events Scale [revised]) at discharge, or during patient follow-up.
- Health-related quality of life or well-being measured by a validated scale (e.g., Quality of Life in Neurological Disorders [Neuro-QoL], EuroQoL-5 Dimensions, 36-Item Short Form Survey [SF-36]) during patient follow-up.
- Neurological complications (neurological deterioration, cerebral vasospasm, delayed cerebral ischaemia, hydrocephalus, rebleeding from a ruptured aneurysm) occurring during the stay.
- Adverse events (haemodynamic instability, increase in intracranial pressure, falls, accidental dislodgement of lines such as extra-ventricular or lumbar drain) related to mobilization.

### Selection of studies

A 4-stage screening process was used to select relevant studies. In the first stage, citations retrieved by the searches were exported to reference management software for screening and duplicate removal (11). In the second stage, 1 reviewer (AF) screened all titles for eligibility. In the third stage, titles and abstracts were independently evaluated by 2 reviewers (AF and FM). In the fourth stage, the full text of each potentially eligible study was assessed by 2 reviewers (AF and FM). Disagreements were resolved by discussion.

### Data extraction and quality assessment

Two reviewers independently performed data extraction using a standardized data extraction form. The Plotdigitizer software (https://plotdigitizer.com/) was used to estimate numerical results when results were presented only as figures (12). Authors of eligible studies were contacted for clarification of methodology and results in the case of unpublished or missing data. One of the included studies was co-authored by 3 of the authors on this paper; therefore, 2 external reviewers completed data extraction and quality assessment using the same standardized data extraction form (13).

Two authors (AF and FM) independently assessed study quality using the Cochrane risk of bias tool for the RCTs and an adapted version of the Newcastle-Ottawa scale for observational studies (14). Any other potential bias was also reported. Any discrepancies in assessments of risk of bias were resolved with consensus.

### Data synthesis and analysis

When sufficient data were available, meta-analysis was performed. We considered studies where early mobilization was initiated within 7 days after SAH onset. One study considered a 2-week delay and was therefore excluded from the meta-analysis (15). For dichotomous variables, a pooled odds ratio with 95% confidence intervals (CI) was calculated. For continuous variables a pooled estimate of mean difference (MD) with 95% CI was calculated. All the meta-analyses included a random effect to account for between-study statistical heterogeneity. Authors were contacted to collect parametric data (means and standard deviations) when missing. For the adverse event rate, a pooled incidence was estimated. This was not possible for device dislodgement and intracranial pressure increase, as the total number of mobilization sessions during which a patient wore such a device was not reported.

The I^2^ statistic was estimated to evaluate the amount of between-study heterogeneity and Cochran’s Q test was performed to assess its statistical significance. Given the low number of studies for each meta-analysis, we were unable to assess the risk of publication bias. All meta-analysis was conducted using R version 4.2.0 (2022-04-22 ucrt), meta package version 7.0.1 (R Foundation for Statistical Computing, Vienna, Austria). When there were insufficient data to perform a meta-analysis, the results of the included studies were reported descriptively.

For both meta-analysis and descriptive reporting of results, when there were more than 2 groups, pooling was performed to compare the group with the earliest mobilization initiation or highest mobilization level with the others. For descriptive reporting, means and standard deviations were recalculated before performing Student’s *t*-test. For categorical data, proportions were summed, and Fisher’s or a χ^2^ test was performed.

## RESULTS

### Flow of trials through the review

**Fig. 1:**
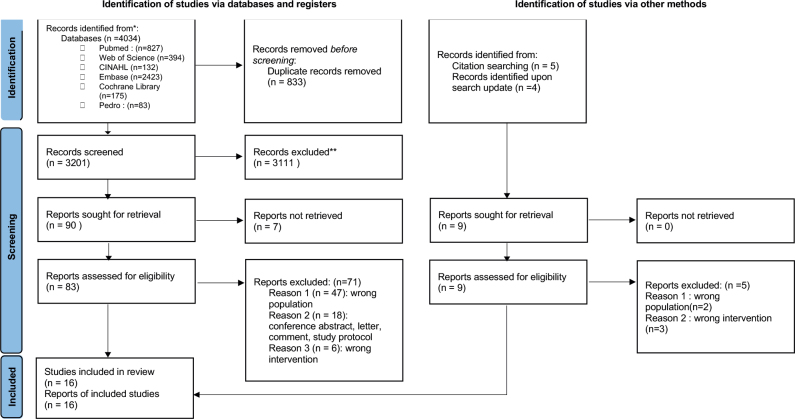
Flow of studies through the review. From: Page MJ, McKenzie JE, Bossuyt PM, Boutron I, Hoffmann TC, Mulrow CD, et al. The PRISMA 2020 statement: an updated guideline for reporting systematic reviews. BMJ 2021; 372: n71. https://doi.org/10.1136/bmj.n71

A total of 4,034 citations were identified, with 833 removed during de-duplication, resulting in 3,201 unique citations screened and 83 full-text publications reviewed (Fig. 1). After excluding 71 publications (47 with an inadequate population, 6 with an inadequate intervention, 15 in the form of conference abstracts, 1 letter, 1 commentary, 1 study protocol), we were able to include 12 studies. Four eligible studies were published after the initial screening. No further eligible articles were found from snowballing and reverse snowballing.

### Characteristics of included trials

Characteristics of the 16 included studies are outlined in Table I. The 16 publications included 1 randomized controlled trial, 7 before–after studies (retrospective, prospective, or ambispective), 5 retrospective cohorts, 2 case series, and 1 case-control study. Most studies were conducted in Japan (*n* = 5) and the United States (*n* = 5), with others in Norway (*n* = 3), China (*n* = 1), France (*n* = 1), and Serbia (*n* = 1). All were monocentric except for 1 study conducted in 5 hospitals (16). Most studies investigated the impact of the implementation of an early mobilization programme, the others compared 2 or more different mobilization initiation times or mobilization levels/intensities. The case-control study investigated the association between cerebral vasospasm and early mobilization (16).

**Table I:** Characteristics of included studies

Study (year)	Objectives	Location	Type of study	Setting	Pathology	Total population			Control group			Intervention				Group differences
						Sample size	Age	Good grade/Poor grade SAH ratio	*n*	Male (%)	Description	*n*	Male (%)	Description	Mean time to mobilization	
Foudhaili (2023)	To evaluate the impact of early out-of-bed mobilization of SAH patients on functional independence at 3 months and cerebral vasospasm	France	Retrospective cohort	Surgical intensive care unit, University Hospital	Aneurysmal SAH	179	53 [44–61]	69/31	148	30%	Out-of-bed mobilization > D4	31	36%	Out-of-bed mobilization ≤ D4	≤ D4	Intervention group: lower grade SAH (*p* < 0.05), less EVD (*p* = 0.001), extubated earlier (*p* < 0.01)
Karic (2014), J Disab	To describe the content and feasibility of early rehabilitation adapted to SAH patients	Norway	Case series	Neuro-intermediate ward of the neurosurgical department, University Hospital	Aneurysmal SAH	37	58 [35–74]	89/11	–	–	None	37	29%	A 7-step mobilization algorithm	D3	No differences reported
Karic (2016), J Neurosurg	To assess the impact of early rehabilitation on global functional outcome 1 year after SAH	Norway	Before–after study	Neuro-intermediate ward of the neurosurgical department, University Hospital	Aneurysmal SAH	171	56 [25–81]	80/20	77	36%	Standard care	94	30%	A 7-step mobilization algorithm	D7	No differences reported
Karic (2016), J Rehabil Med	To evaluate the effect of early mobilization and rehabilitation on complications during the acute phase after SAH	Norway	Before–after study	Neuro-intermediate ward of the neurosurgical department, University Hospital	Aneurysmal SAH	168	55 [25–81]	83/17	76	37%	Standard care, not protocolized	92	30%	A 7-step mobilization algorithm	D7.4 in poor-grade; D0.9 in good-grade	No differences reported
Milovanovic (2016)	To assess the impact of early verticalization of SAH patients on early postoperative complications, occurrence of vasospasm and occurrence of motor deficits	Serbia	Randomized controlled trial	Neurosurgery clinic	Aneurysmal SAH, classified as grade I, II, or III on the Hunt and Hess scale	65	53 [25–71]	100/0	34	32%	Verticalization initiated from D12 post bleeding	31	32%	Verticalization initiated on days 2–5 post bleeding	< D5	Intervention group: non-statistically significant tendency for more pneumonia, fever meningitis, wound dehiscence
Moyer (2017)	To assess the feasibility, safety, and outcome of an early mobility protocol for patients with SAH with an EVD	USA	Before–after study	Neuro-ICU, University Hospital	Aneurysmal SAH with EVD	45	58	85/15	19	10%	Strict bedrest while EVD in place	26	15%	Mobilization algorithm	D6.5	No differences reported
Okamura (2021)	To investigate the relationship between early mobilization and home discharge and functional status in patients with SAH	Japan	Retrospective cohort	Hospital	Aneurysmal SAH	35	66 ± 14	63/37	13	36%	Participation in walking rehabilitation > D14	22	39%	Participation in walking rehabilitation ≤ D14	D4.2	Intervention group: lower grade SAH (*p* < 0.001)
Olkowski (2013)	To examine the safety and feasibility of an early mobilization programme for patients with aneurysmal SAH	USA	Before–after study	Neurosurgical ICU, Hospital	Aneurysmal SAH	25	55.6 ± 11.8	88/12	–	–	–	25	24%	Early mobilization programme	D3.2	No differences reported
Olkowski (2015)	To determine the impact of an early mobilization programme for patients with aneurysmal SAH on function and hospital length of stay	USA	Case series	Neurosurgical ICU, Hospital	Aneurysmal SAH	93	54	74/26	38	26%	Standard care	55	36%	Early mobilization programme	D4.2	No differences reported
Riordan (2015)	To investigate the effect of early mobilization using mild exercise in patients with SAH after securization of the aneurysm	USA	Retrospective cohort	University hospital	Aneurysmal SAH	80	56	82/18	58	29%	Fusion of: initiation of active physical therapy within D4–D9, D10–D19, ≥ D20)	22	27%	Initiation of active physical therapy < D4	< D4	Intervention group
Shimamura (2014)	To investigate the effect of early ambulation on outcome in advanced-age SAH	Japan	Retrospective cohort	Department of Neurosurgery, University Hospital	Aneurysmal SAH, age > 70 years old	71	76 [70–87]	100/0	45	ns	Fusion of: ambulation within D6–D10, D11–D15, < D15	26	ns	Ambulation < D6	< D6	No differences reported
Takara (2022)	To investigate the association between initiating mobilization within 7 days after onset and symptomatic cerebral vasospasm in patients with aneurysmal SAH	Japan	Case control study	Five hospitals	Aneurysmal SAH	510	< 65: 53% ≥ 65: 47%	70/30	453	29%	No symptomatic cerebral vasospasm group	57	26%	Symptomatic cerebral vasospasm group	NA	No SCV group: lower grade SAH (*p* < 0.01)
Uemura (2018)	To investigate the impact of sitting position within 13 days after surgery and the level of activities of daily living and discharge destination in SAH patients	Japan	Retrospective cohort	Department of Neurosurgery, University Hospital	Aneurysmal SAH, middle-aged and elderly patients	43	67	51/49	21	19%	Fusion of: DA group: bedrest until D13; DS group: bedrest with head-up position until D13	22	14%	Early sitting group: sitting out-of-bed < D13	< D13	Intervention group: more coiling operation (*p* < 0.05), lower NIHSS score (*p* < 0.05)
Yang (2023)	To evaluate the validity, safety, and feasibility of ICF-based early progressive mobilization in patients with severe aneurysmal SAH	China	Before–after study	Neurosurgical ICU, University Hospital	Aneurysmal SAH patients, Hunt and Hess III–IV, mechanically ventilated > 48 h	68	69	63/37	34	41%	Passive joint movement	34	32%	ICF-based early progressive mobilization	D3	No differences reported
Yokobatake (2022)	To investigate the impact of early rehabilitation for patients with aneurysmal SAH on the occurrence of complications, disuse complications, and independence at 90 days post-onset	Japan	Before–after study	Tertiary hospital centre	aneurysmal SAH	111	68 [56–80]	57/43	55	31%	Standard care	56	29%	Early rehabilitation programme	D5	Intervention group: more endovascular treatment (*p* < 0.001)
Young (2019)	To determine whether a nurse-driven mobilization protocol would result in safe and more frequent mobilization than institutional standard care.	USA	Before–after study	Neuro-ICU, University Hospital	SAH with EVD	32	56 [33–90]	88/12	15	31%	2 groups: standard care, and PT/OT-driven mobilization protocol	17	53%	Nurse-driven mobilization protocol	D4.9	No differences reported

EVD: external ventricular derivation; ICU: intensive care unit; SCV: symptomatic cerebral vasospasm.

Three studies were part of the same project and their populations overlapped, but as they assessed different outcomes we decided to include all 3 of them (17–19). Two studies were carried out by the same team, with no overlapping populations, and both were included (20, 21). Two other studies were carried out by the same team. The first compared a mobilization protocol led by physiotherapists (PT) and occupational therapists (OT) with standard care and was included as is (22). The second compared a nurse-driven protocol with both standard care and the PT/OT-driven protocol (23). As the authors did not report a difference between the time to initiation of the PT/OT protocol and the nurse-led protocol, only the comparison of the nurse-led protocol with standard care was reported in this study.

Three studies involved more than 2 groups with different mobilization initiation times (24, 25), or different mobilization levels (15). Whenever possible group pooling was performed as described in the Methods section.

### Quality assessment of included studies

**Fig. 2:**
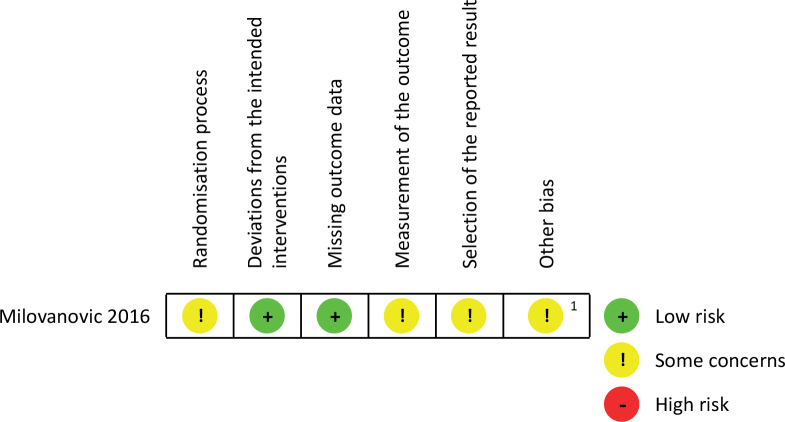
Risk of bias assessment of randomized, controlled trials. It is uncertain whether the study was sufficiently powered and the statistical analyses adequate. ^1^Very little detail given regarding the mobilization protocol, including safety assessment for initiating mobilization and cancellation criteria.

The risk of bias of the RCT was moderate (Fig. 2). The method of randomization and/or concealment was not clearly described, and blinding of assessors was unlikely. The study protocol was neither available in a trial registry nor published. It is uncertain whether the study was sufficiently powered and the statistical analyses adequate. No details were given regarding the mobilization protocol safety criteria and patient screening.

Assessment of the 15 other studies demonstrated a low to moderate risk of bias (Tables II and III). Most studies were representative of patients with SAH receiving mobilization and ascertainment of exposure to mobilization was described in all studies. Some of the retrospective studies reported significant between-group differences in the severity of SAH, with patients who were mobilized early being less severe. Most studies performed adjustments for confounders in their analysis.

**Table II:** Risk of bias assessment of cohorts

Cohort								
Study	Selection				Comparability	Outcome		

	Representativeness of the exposed cohort	Selection of the non-exposed cohort	Ascertainment of exposure	Outcome was not present at start	Comparability of cohorts	Assessment of outcome	Follow-up long enough for outcomes to occur	Adequacy of follow up of cohorts
Foudhaili, 2023	*	*	*	*	*	*	*	*
Karic, Disability and Rehabilitation, 2014	*	NA	*	*	NA	*	*	NA
Karic, Journal of Neurosurgery, 2016	*	*	*	*	*	*	*	*
Karic, Journal of Rehabilitation Medicine, 2016	*	*	*	*	*	*	*	*
Moyer, 2017	*	NA	*	*	NA	*	*	NA
Okamura, 2021	*	*	*	*	*	*	*	*
Olkowski, Physical Therapy, 2013	*	NA	*	*	NA	*	*	NA
Olkowski, Journal of Acute Care Physical Therapy, 2015	*	*	*	*	*	*	*	*
Riordan, 2015	*	*	*	*	*	*	*	*
Shimamura, 2014	*	*	*	*	*	*	*	*
Uemura, 2018	*	*	*	*	*	*	*	*
Yang, 2023	*	*	*	*	*	*	*	*
Yokobatake, 2022	*	*	*	*	*	*	*	*
Young, 2019	*	NA	*	*	NA	*	*	*

NA: not applicable.

**Table III:** Risk of bias assessment of case-control study

Reference	Case control							
	Selection			Comparability		Exposure		

	Adequate definition of patient cases	Representativeness of patient cases	Selection of controls	Definition of controls	Comparability of cases and controls	Ascertainment of exposure	Same ascertainment method for participants	Non-response rate
Takara, 2022	*	*	*	*	*	*	*	*

### Characteristics of the participants

The studies included a total of 1,733 patients, and study cohorts ranged from 25 to 510 patients (median 70). Key characteristics of participants are outlined in Table I. The mean age of participants ranged from 52 to 76 years. Most patients were women. Thirteen of 16 studies (81%) included patients with SAH of any grade, 2 studies excluded patients with poor-grade SAH (24, 26) and 1 excluded patients with Hunt and Hess I and II SAH (27). Overall, 15 out of 16 studies had a good grade to poor grade SAH ratio in favour of good grade, with the majority of studies including more than 70% of patients with good grade SAH. The last study had a good-grade/poor-grade ratio of 51/49(15). Fourteen of 16 studies (90%) included patients with SAH of any age; the remaining studies included middle-aged and elderly patients (15, 24).

### Characteristics of the mobilization strategies

There was heterogeneity in multiple aspects of the interventions throughout the 16 included studies.

Nine studies evaluated mobilization algorithms or programmes (Table IV) (17–23, 27, 28). The timing for initiating mobilization, when reported, did not exceed 24 h after the aneurysm repair or admission (17–21, 23, 28).

**Table IV:** Early mobilization protocols modalities of included studies

	Karic (2014) and Karic (2016), J Rehabil Med & Karic (2016), J Neurosurg	Moyer (2017)	Olkowski (2013) and Olkowski (2015)	Yang (2023)	Yokobatake (2022)	Young (2019)
Timing for initiating mobilization	≤ 24 h after the aneurysm repair	Not specified	≤ 24 h after admission	Not specified	As soon as possible after aneurysm repair	After a 6-h post-repair flat bedrest restriction
Criteria for initiating mobilization	No severe vasospasm Monitoring parameters within defined limits (see below)	Tolerance to 30 min of drain clamping ICP < 20 mmHg Stable neurological examination	Lindegaard ratio ≤ 3.0 or middle cerebral artery MFV ≤ 120 cm/s MAP ≥ 80 and ≤ 110 mmHg HR ≥ 40 and ≤ 130 bpm RR ≥ 40 breaths/min SPO2 ≥ 88% ICP ≤ 15 mmHg No evidence of seizure activity Stable neurological examination Able to open eyes in response to voice Ability to move one extremity on command	No symptomatic cerebral vasospasm or new neurologic events	Stable neurologic examination Stable imaging No symptomatic cerebral vasospasm	Tolerance to 30 min of drain clamping ICP < 20 No symptomatic vasospasm EVD securely fixed Relative mobilization exclusion criteria: fluctuating neurological examination, pulmonary or cardiovascular instability, and patient refusal
Criteria for stopping mobilization	MAP < 80 mmHg CPP < 70 or 90 mmHg in the presence of vasospasm ICP > 20 mmHg SPO2 < 95% CO2 pressure < 3.5 or > 6 HR < 40 or > 100 RR< 12 or > 20 Neurological deterioration Patient discomfort	ICP > 20mmHg Intolerance to activity signs/symptoms including: ° Acute change in mental status ° Acute headache ° Acute focal/or worsening of deficits ° Nausea/vomiting HR, BP and SPO2 are monitored, but no threshold is indicated for mobilization cessation	MAP < 70 and > 120 mmHg HR < 40 or > 130 bpm RR < 5 or > 40 breaths/min SPO2 < 88% ICP > 15 mmHg Fall Acute change in neurological presentation	HR variation > 20% from the resting level BP variation ≥ 20 mmHg RR > 40 breaths/min, or < 5 breaths/min SPO2 < 88% and longer than 3 min Not following the directions in case of abnormal mental status Intolerance due to discomfort, diaphoresis, or trembling Malignant arrhythmia	Worsening neurological symptoms Decreased consciousness level SBP > 160 mmHg or drop ≥ 30 mmHg Heart rate fluctuations Oxygenation fluctuations Respiratory frequency fluctuations	Acute change in mental status Acute onset of headache Acute focal or worsening of deficits Significant increase of ICP > 20 mmHg
Action to be taken in presence of stopping criteria	Return to the previous step if tolerated	Interruption of all PT/OT treatment Return to supine position with head of bed elevated	Interruption of mobilization	Return to the previous step if tolerated	Interruption of mobilization	Return to the previous step if tolerated
Description of the mobilization programme	Seven-step algorithm. (0) bed rest with 30° head elevation (1) bed rest with 60° head elevation (2) bed rest with 80° head elevation (3) sitting one the edge of the bed (4) sitting in the chair (5) standing at bedside (6) go to the toilet and into the hallway Minimum one-day delay between steps 0, 1, and 2 No minimum delay for subsequent steps Other components: passives and active exercises, ADL training, balance training Frequency: 5 days/week by the rehabilitation team, continued in evenings and at weekends by nurses Mobilization programme is part of a wider rehabilitation strategy that will not be described here	Prior to mobilization: EVD clamping and checking of EVD fixation and intact dressing by the nurse First step is sitting at the edge of the bed Subsequent activities include standing, side-to-side stepping and performing basic ADLs After mobilization: EVD set back to pre-session level and reopened Frequency: 7 days/week	Progressive programme: (0) bed mobility training in supine position (1) supine with 30° head of bed elevation (passive and/or active ROM) (2) sitting on the edge of the bed (passive and/or active ROM) (3) out-of-bed activities (standing training, balance, posture, ADL training, gait training, sitting out of bed) Duration of sessions: 30 to 60 minutes. Duration of sitting out of bed: as long as the patient remained stable and tolerant Frequency: 7 days/week	4 levels of mobilization: (1) PROM + passive cycle (15 min, no resistance) HOB elevated in 3 steps (each step: ≥ 1 h/day, ≥ 1 day before moving to the next step if tolerated) (2) AROM, active cycle (20 min, no resistance) Bridge (≥ 3 per session), rolling, sitting on the edge of the bed, watching video (3) Strength training, functional exercise, active cycle (20 min, resistance ≥ 1), bridge (≥ 8 per session), transferring from supine to sitting, sitting on the edge of bed, watching video (4) Strength training, functional exercise, active cycle (20 min, resistance ≥ 1), OOB exercise (sitting on the bedside chair, sit to stand, standing on the bedside, marching on the spot, walking alongside the bed) Frequency: 1/day, 5 days/week	Depending on the SAH severity: – Active mobilization and walking training – ADL support – Passive sitting position Gradual increase of the programme according to patient tolerance Duration of sessions: 20 to 40 min Dose depends on SAH severity. Frequency: twice a day	4 levels: (1) Mobilization at edge of bed (optional) (2) Lift to bedside chair (3) Stand pivot to bedside chair/ perform ADL with assistance (4) Ambulation Intensity and duration of activity: subject to patient preference, tolerance, and nursing workflow Duration of sitting out of bed: maximum 3 h Frequency: 7 days/week
Composition of the staff involved	PT, OT, RN	PT, OT, RN	PT, OT, RN	PT	PT, OT, RN	PT, OT, RN

ADL: activities of daily living; AROM: active range of motion; BP: blood pressure; CPP: cerebral perfusion pressure; HOB: head of bed; HR: heart rate; ICP: intracranial pressure; MAP: mean arterial pressure; MFV: mean flow velocity; OOB: out-of-bed; OT: occupational therapist; PROM: passive range of motion; PT: physiotherapist; RN: registered nurse; RR: respiratory rate; SBP: systolic blood pressure; SPO2: peripheral oxygen saturation.

Mobilization always began only after the aneurysm had been secured, or in the absence of an underlying aneurysm. Daily assessment for initiating mobilization included the absence of symptomatic cerebral vasospasm, stability of haemodynamic and respiratory parameters, no intracranial hypertension, and stable neurological examination or imaging. Ischaemia, rebleeding, new neurological symptoms, or fluctuations in neurological examination led to a pause or setback in the mobilization programmes. In studies evaluating patients with external ventricular drain (EVD), tolerance to EVD clamping was also required, as well as verification that the EVD was securely fixed prior to mobilization.

The starting level of exercise varied from in-bed exercises to edge-of-bed sitting and mobilization progressed once the previous step had been well tolerated. All studies defined monitoring parameters and criteria for stopping mobilization mostly including haemodynamic and respiratory instability, an increase of intracranial pressure (ICP), acute change in neurological examination, and signs of intolerance (nausea, vomiting, headache). Mobilization steps, frequency, duration, and intensity varied greatly across studies.

Mobilization involved physiotherapists in all studies, and most of the time occupational therapists and/or nurses. Two studies indicated that staff must be qualified and/or trained (22, 28), and 1 study specified that physiotherapists must have more than 3 years’ experience in critical care (27).

In the cohort studies, case-control study, and randomized controlled trial, the mobilization modalities and timeframe defining the early mobilization group varied widely.

### Effects of early mobilization on physical function and physical performance tasks in patients with subarachnoid haemorrhage

**Fig. 3:**
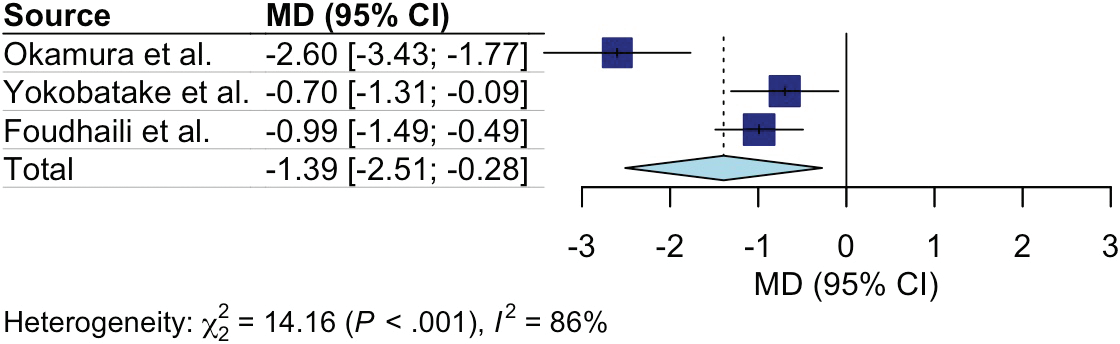
Forest plot of effect of early mobilization on physical function at discharge. CI: confidence interval; MD: mean difference.

**Fig. 4:**
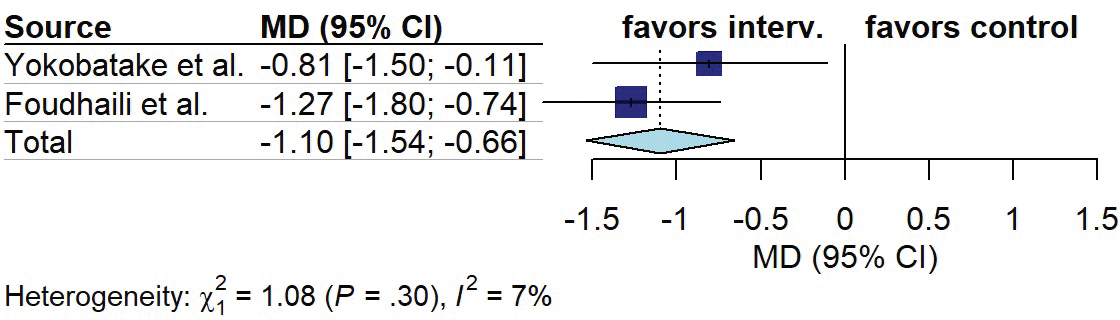
Forest plot of effect of early mobilization on physical function at 3 months.

Six out of 16 studies reported measures of physical function or physical performance tasks (13, 15, 18, 24, 28, 29). The timeframes and outcomes reported were diverse (Table V). Three studies reported functional status at ICU discharge assessed by modified Rankin Scale (mRS) score (13, 28, 29). Pooled analysis demonstrated a significant improvement in functional status favouring early mobilization (MD –1.39, 95% CI –2.51 to –0.28, I^2^ = 86%) (Fig. 3). Two studies reported 3 months functional status assessed by mRS score (13, 28). Pooled analysis demonstrated a significant improvement in functional status favouring early mobilization (MD –1.10, 95% CI –1.54 to –0.66, I^2^ = 7%) (Fig. 4).

**Table V:** Effects of mobilization on physical function and physical performance tasks

Study (year)	Outcome measure	Timepoint	Mean ± standard deviation, median (IQR) or *n* (%)					Comments
			Intervention	*n*	Comparator	*n*	*p*-value	
Okamura (2021)	AI	Discharge	1.9 ± 1.8	13	6.7 ± 2.5	22	**< 0.001**	Ambulatory index: 0 = Asymptomatic; Fully active; 9 = Restricted to wheelchair; unable to transfer self independently
Yokobatake (2022)	Barthel Index	Discharge	63 (14–100)	56	40 (5–70)	55	**0.03**	Barthel index: 0 = fully dependent; 100 = fully independent
Okamura (2021)	FAC	Discharge	4.2 ± 0.9	13	1.2 ± 1.5	22	**< 0.001**	Functional ambulation classification: 0 = absolute inability to walk even with external help; 5 = normal deambulation
Yokobatake (2022)	FAC	Discharge	3 (1–4)	56	2 (0–4)	55	**0.04**	Functional ambulation classification: 0 = absolute inability to walk even with external help; 5 = normal deambulation
Okamura (2021)	GOS	Discharge	4.5 ± 0.7	13	3.0 ± 0.6	22	**< 0.001**	GOS: 1 = dead; 5 = good recovery
Uemura (2018)	Motor FIM efficacy	Discharge	1.93 ± 1.13	22	0,73 ± 0.94	21	**< 0.001**	Motor FIM efficacy = discharge motor FIM – 4th day motor FIM
Okamura (2021)	mRS	Discharge	1.5 ± 1.4	13	4.1 ± 0.8	22	**< 0.001**	Modified Rankin Scale: 0 = fully independent; 6 = death
Yokobatake (2022)	mRS	Discharge	3 (1–4)	56	4 (2–5)	55	**0.02**	Modified Rankin Scale: 0 = fully independent; 6 = death
Foudhaili (2023)	mRS	Discharge	2 (1–3)	31	3 (1–4)	148	**0.007**	Modified Rankin Scale: 0 = fully independent; 6 = death
Shimamura (2014)	GOS: GOS > 3	30 days	23 (88%)	26	24 (53%)	45	**0.0036**	GOS: 1 = dead; 5 = good recovery
Yokobatake (2022)	Barthel Index	90 days	98 (40–100)	56	70 (5–100)	55	**0.048**	Barthel index: 0 = fully dependent; 100 = fully independent
Yokobatake (2022)	FAC	90 days	5 (2–5)	56	3 (1–5)	55	**0.03**	Functional ambulation classification: 0 = absolute inability to walk even with external help; 5 = normal deambulation
Foudhaili (2023)	mRS: mRS < 3	90 days	26 (84%)	31	83 (56%)	148	**0.004**	Modified Rankin Scale: 0 = fully independent; 6 = death
Yokobatake (2022)	mRS	90 days	2 (0–4)	56	3 (1–5)	55	**0.01**	Modified Rankin Scale: 0 = fully independent; 6 = death
Foudhaili (2023)	mRS	90 days	1 (0–2)	31	2 (1–4)	148	**0.004**	Modified Rankin Scale: 0 = fully independent; 6 = death

AI: ambulatory index; FAC: functional ambulation classification; FIM: functional independence measure; GOS: Glasgow outcome scale; mRS: modified Rankin Scale. Bold values mean statistical signicance (*p* < 0.05).

### Effects of early mobilization on cognitive function in patients with subarachnoid haemorrhage

Three out of 16 studies reported measures of cognitive function (15, 24, 26). The outcomes and timeframes reported varied (Table VI). Meta-analysis could not be performed.

**Table VI:** Effects of mobilization on cognitive function and performances

Study (year)	Outcome measure	Timepoint	Mean ± standard deviation, median (IQR) or *n* (%)					Comments
			Intervention	*n*	Comparator	*n*	*p*-value	
Milovanovic (2016)	MMSE No cognitive problems: MMSE > 24	Discharge	14 (41.2%)	34	19 (61.3%)	31	**0.04**	
Uemura (2018)	Cognitive FIM efficacy	Discharge	1.64 ± 1.25	22	0.46 ± 1.36	21	0.46	Cognitive FIM efficacy = discharge cognitive FIM 4th day cognitive FIM
Milovanovic (2016)	MMSE No cognitive problems: MMSE > = 24	30 days	22 (64.7%)	34	26 (83.9%)	31	**0.025**	
Shimamura (2014)	Revised-Hasegawa Dementia Scale No dementia: HDS-R > 20	30 days	16 (61.5%)	26	11 (24.4%)	45	**0.019**	Highest HDS-R score is 30 and HDS-R threshold is 21. Three categories of dementia were established: severe (0–10), moderate (11–20), and normal (21–30)
Milovanovic (2016)	MMSE No cognitive problems: MMSE > 24	90 days	28 (82.4%)	34	28 (90.3%)	31	0.073	

HDS-R: Hasegawa Dementia Scale; MMSE: Mini Mental State Examination.Bold values mean statistical signicance (*p* < 0.05).

The RCT found a significant decrease in cognitive function in the early verticalization group at discharge and 1 month but no difference at 3 months using the MMSE score (26). One study found no difference in cognitive function at discharge using the cognitive FIM (15). The last study focused on elderly patients and reported that early ambulation was associated with a decrease in the rate of dementia at 1 month using the Revised-Hasegawa Dementia Scale (24).

### Effects of early mobilization on psychological status in patients with subarachnoid haemorrhage

Only the RCT reported psychological outcomes (26). Meta-analysis could not be performed.

A significant increase in depression rate in the early verticalization group was found at discharge, 1 month, and 3 months using the Zung scale for self-rating depression. A significant increase in anxiety rate was found at 1 month and 3 months using the Zung scale for self-rating anxiety. No between-group difference was found at discharge.

### Effects of early mobilization on neurological complications in patients with subarachnoid haemorrhage

**Fig. 5:**
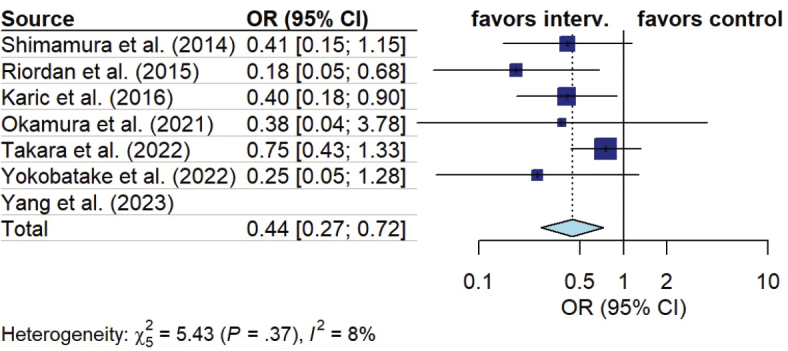
Forest plot of effect of early mobilization on clinical vasospasm.

**Fig. 6:**
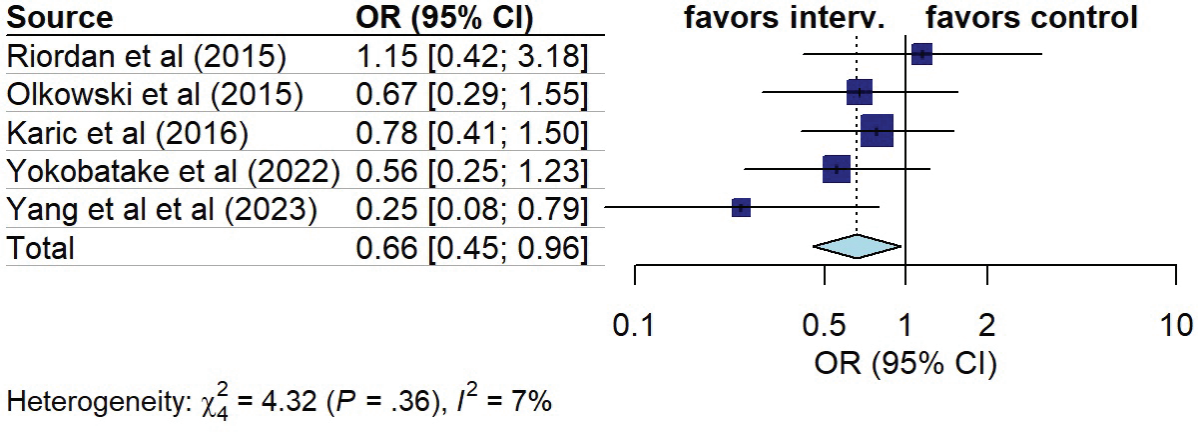
Forest plot of effect of early mobilization on radiological vasospasm.

*Cerebral vasospasm.* Eleven out of 16 studies assessed the impact of early mobilization on cerebral vasospasm occurrence. The studies either reported the total number of vasospasms (13, 21) and/or differentiated between symptomatic and asymptomatic vasospasms (19, 25, 27), only reported symptomatic vasospasms (16, 24, 28, 29), or did not specify (15, 26). Seven studies reported clinical or symptomatic vasospasms (16, 18, 24, 25, 27–29). In a pooled analysis, patients in the early mobilization group had a lower probability of presenting symptomatic vasospasm (OR 0.44, 95% CI 0.27 to 0.72, I^2^ = 8%) (Fig. 5). Five studies reported radiographic vasospasm (18, 21, 25, 27, 28). In a pooled analysis, patients in the early mobilization group had a lower probability of presenting radiographic vasospasm (OR 0.66, 95% CI 0.45 to 0.96, I^2^ = 7%) (Fig. 6).

**Fig. 7:**
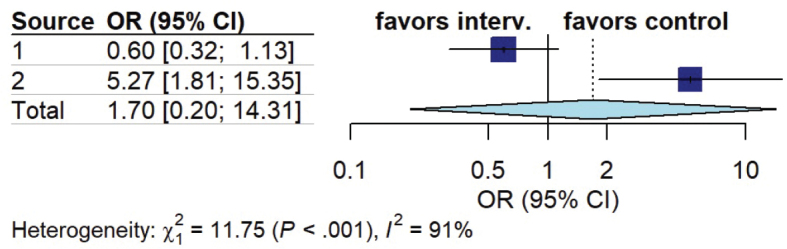
Forest plot of effect of early mobilization on cerebral ischaemia.

*Delayed cerebral ischemia.* Two out of 16 studies assessed the impact of early mobilization on cerebral ischaemia rate. Pooled analysis demonstrated no significant difference between the 2 groups (OR 1.70, 95% CI 0.20 to 14.31, I^2^ = 91%) (Fig. 7).

**Fig. 8:**
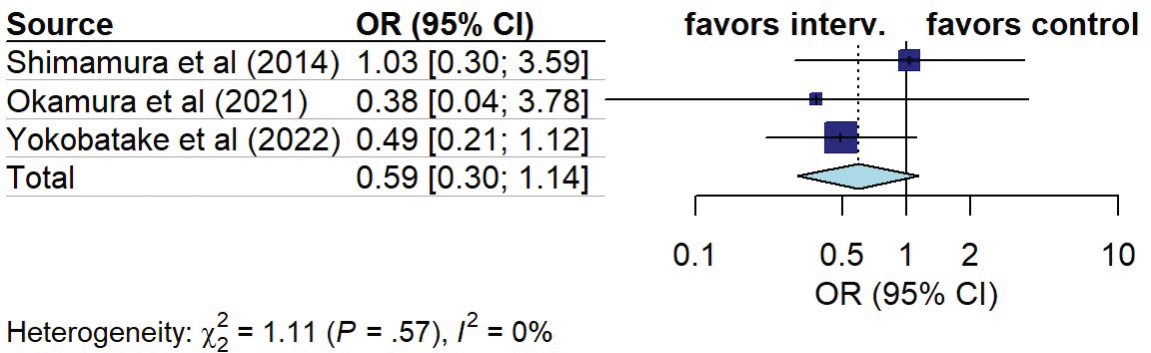
Forest plot of effect of early mobilization on hydrocephalus.

*Hydrocephalus.* Four out of 16 studies assessed the impact of early mobilization on hydrocephalus rate, 3 of which were included in the meta-analysis. Pooled analysis demonstrated no significant difference between the 2 groups (OR 0.59, 95% CI 0.30 to 1.14, I^2^ = 0%) (Fig. 8).

*Rebleeding after aneurysm repair.* Three out of 16 studies assessed the impact of early mobilization on rebleeding occurrence (Table VII) (19, 28, 29). Due to the limited number of events in both groups, no meta-analysis was performed.

**Table VII:** Rebleeding events

Study (year)	Intervention	*n*	Comparator	*n*	*p*-value
Karic (2016), J Neurosurg	0 (0%)	94	0 (0%)	77	**> 0.05**
Okamura (2021)	0 (0%)	13	0 (0%)	22	**> 0.05**
Yokobatake (2022)	1 (2%)	56	0 (0%)	55	**> 0.05**

Bold values mean statistical signicance (*p* < 0.05).

### Adverse events related to early mobilization

**Fig. 9:**
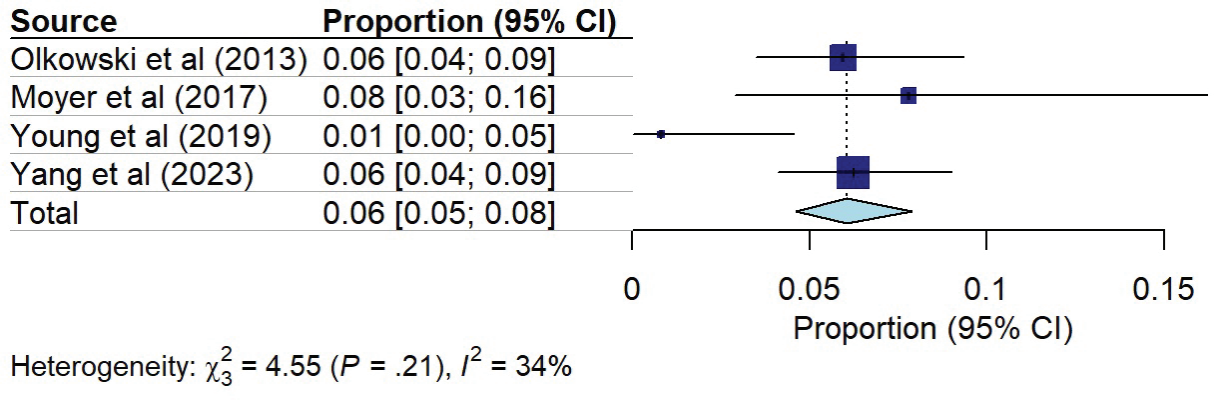
Pooled incidence of adverse events related to mobilization.

Six studies examined the safety of early mobilization (17, 19, 20, 22, 23, 27), of which 4 reported the number of mobilization sessions (20, 22, 23, 27). They included a total of 134 patients and 900 mobilization sessions with 24 reported safety events, for a cumulative incidence of 6% (Fig. 9).

Haemodynamic changes were the most reported type of safety event (*n* = 21; 2.3%). They consisted of 11 hypotensive episodes (MAP < 70 mmHg, 2 without definition), 7 hypertensive episodes (MAP > 120 mm Hg), and 3 heart rate changes (1 HR > 130 bpm, 2 HR changes > 20%) (20, 22, 27). Two respiratory events (0.2%) and 1 neurologic change (0.1%) were reported (22). No fall was reported during the 900 mobilization sessions.

For the events that could not be meta-analysed, the number of events reported were: 9 non-invasive line removals, 4 invasive line removals, 4 increases in ICP, and 9 other events (pain, fatigue, headache, drain malfunction, tracheostomy tube out of midline, feeding tube unfasten). Regarding the ICP increase, 2 of the 4 events were reported as not being associated with a decrease in cerebral perfusion pressure below 60 mmHg (27). No details were given for the 2 remaining events (22, 23).

### Effects of early mobilization on health-related quality of life or well-being in patients with subarachnoid haemorrhage

None of the included studies reported the impact of early mobilization on health-related quality of life or well-being.

## DISCUSSION

In this systematic review and meta-analysis, we found that the implementation of early mobilization in patients with subarachnoid haemorrhage could be associated with better clinical outcomes including improved functional status assessed by mRS score at hospital discharge and at 3 months. Early mobilization was also associated with a decrease in both radiological and clinical vasospasm rates without changes in the rate of cerebral ischaemia. Adverse events occurred in 6% of mobilization sessions, the majority of which were minor haemodynamic changes. These results suggest that early mobilization could be associated with improved outcomes in SAH patients and should therefore be adequately tested in further interventional trials.

Early mobilization of intensive care patients is now widely recommended and integrated into the standard of care (30). It has shown numerous beneficial effects on physical and cognitive functions (31–33). However, results are sometimes discordant, with negative trials and concerns over safety (34). Optimal timing and modalities have yet to be defined. In the case of brain-injured patients, the safety concerns are even greater and addressing the timing, intensity, and frequency of mobilization is crucial. Indeed, the results of the AVERT trial really called into question the very early mobilization in brain-injured patients (35). Early mobilization in SAH patients must balance the potential benefits of enhancing neuroplasticity and functional recovery with the risks of exacerbating vasospasm, delayed cerebral ischaemia, and increasing the ischaemic penumbra zone (36). However, the 2023 AHA/ASA recommendations called for the implementation of an early mobilization protocol for patients with SAH to improve functional outcome (8). The level of recommendation is moderate, and the modalities of mobilization were not specified (8). Practice surveys have shown that mobilization is highly heterogeneous, ranging from prolonged bedrest to early ambulation, and limited by several safety concerns (13, 37, 38). In this review, the mobilization strategies were secured by investigators with daily verification of eligibility criteria for mobilization, monitoring of clinical and physiological parameters, and progressiveness. This is consistent with the assumption of Paton et al. that intensive, maximalist mobilization could generate a higher rate of adverse events than progressive mobilization (39).

In this review, adverse events were reported in 6% of mobilization sessions. In the general ICU population, 2 meta-analyses reported an adverse event rate of less than 3% (14, 39). It is difficult to compare these rates, due to the lack of clarity in the definition of adverse events in the present included studies. It can be assumed that more haemodynamic changes are expected in neurocritical patients, especially as they are often confined to bed for several days, favouring sympathetic system maladaptation and orthostatic intolerance (40, 41). However, the clinical impact of these changes remains to be evaluated. The evaluation of adverse events in the context of early and increased mobilization is difficult; increasing mobilization will likely increase mobilization-related adverse events (42). Nevertheless, this must be weighed against the impact of mobilization on functional results and the multiple complications of bed rest, such as thromboembolic events, pressure sores, neuromuscular, respiratory, and cardiovascular complications (43).

Among the safety concerns reported in the aforementioned surveys – and therefore obstacles to mobilization – were the presence of vasospasm and the fear that mobilization would generate or promote it (37, 38, 44). Interestingly, the present review did not find any protocol contraindicating mobilization in the presence of asymptomatic vasospasm. Combined with the fact that early mobilization was associated with a reduction in the frequency of vasospasm, these results could help to overcome these barriers.

This meta-analysis is to our knowledge the first to suggest that early mobilization in patients with SAH is associated with lower rates of radiological and clinical vasospasm and improved functional status at discharge and at 3 months. Two systematic reviews examining the mobilization of patients with SAH were identified. The first, a Cochrane review, targeted randomized controlled trials including patients with unsecured aneurysms, and found no eligible studies (45). The second, published in 2023, by Morello et al. had inclusion criteria similar to those used in the present study (46). They found no difference in functional status assessed by mRS score at hospital discharge (46). The contrast between our results is potentially attributable to an error in the reporting of results from Foudhaili et al. showing improved functional status in the early mobilization group (13). Morello et al. showed a significant decrease in radiographic vasospasm rate, whereas no difference was found in clinical vasospasm. The difference in our results could stem from the inclusion by Morello et al. of the negative randomized controlled trial that we chose to exclude in the absence of a clear definition of vasospasm (26) They also studied adverse events, defined as mortality, pneumonia, and thrombosis rate, and did not find any between-group differences (46). Last, they showed a significant decrease in hospital length of stay, which suggests that early mobilization in SAH patients could have economic benefits (46).

### Limitations

The present review presents several limitations. As previously stated, the definition of early mobilization is still highly variable (47). In this review, studies were included if the intervention group had mobilization occurring earlier than the comparator. Our meta-analysis focused on interventions initiated within the first 7 days after SAH onset. In the ICU general population, early mobilization is commonly defined as initiated within the first 72 h (48). It is therefore debatable whether these interventions can be described as early or not. However, it should be borne in mind that neurological populations may present additional characteristics that initially hinder mobilization, compared with the general population (49). Interestingly, the 9 studies that involved a mobilization protocol initiated mobilization within 24 h after ICU admission or aneurysm repair, similar to practices in the general ICU population (31, 32).

Regarding the quality of the included studies, our review included only 1 randomized controlled trial, with moderate risk of bias, and uncontrolled cohorts or historical control group trials with low to moderate risk of bias. Regarding the randomized controlled trial, in addition to the biases assessed by the Cochrane risk of bias tool, the statistical power and quality of the statistical analyses are questionable. In addition, unusual characteristics were found in their population, such as a high frequency of postoperative meningitis. The description of the medical management and mobilization procedure was also very succinct, and the latter did not seem to include any of the safety measures described above. This may have contributed to it being the only negative trial in this review. Hence, its contribution to the body of evidence should be interpreted cautiously. More generally, most trials had small sample sizes and were monocentric. There was also considerable heterogeneity in mobilization procedures, whether in terms of time to initiation, type of mobilization, or dosage. The AVERT results suggest that dosage and time to first mobilization play a crucial role in achieving favourable functional outcome (9). These parameters therefore need to be determined as part of future clinical trials and clearly described to ensure their reproducibility and enable their inclusion in future clinical guidelines. There was also significant heterogeneity in the judgement criteria used, which limited the number of studies included in each meta-analysis. The results of this meta-analysis should therefore be viewed with caution.

Last, we were not able to assess the effect of early mobilization on cognitive status and quality of life of patients with SAH due to a lack of data. Despite functional recovery, patients with SAH report cognitive impairment, anxiety, depression, activity limitations, and impaired quality of life (50, 51). The mRS score correlates poorly with these parameters (51). Future clinical trials should use appropriate scales to assess the impact of early mobilization on cognitive and psychological status and quality of life.

### Conclusion

This systematic review and meta-analysis found that the implementation of early mobilization in patients with subarachnoid haemorrhage was associated with better clinical outcomes including improved functional status and decreased rate of cerebral vasospasm. The rate of adverse events occurring during mobilization was around 6% in the early mobilization group, mainly mild events. These results suggest that early mobilization could be associated with improved outcomes in SAH patients and should therefore be adequately tested in interventional trials.

## Supplementary Material

EARLY MOBILIZATION IN PATIENTS WITH ANEURYSMAL SUBARACHNOID HAEMORRHAGE MAY IMPROVE FUNCTIONAL STATUS AND REDUCE CEREBRAL VASOSPASM RATE: A SYSTEMATIC REVIEW WITH META-ANALYSIS
