# Supplementary material for: EARLY MOBILIZATION IN PATIENTS WITH ANEURYSMAL SUBARACHNOID HAEMORRHAGE MAY IMPROVE FUNCTIONAL STATUS AND REDUCE CEREBRAL VASOSPASM RATE: A SYSTEMATIC REVIEW WITH META-ANALYSIS
Source: J Rehabil Med. 2024 Oct 18;56:41225. doi: 10.2340/jrm.v56.41225 (PMC11497628; doi:10.2340/jrm.v56.41225)
Supplement: EARLY MOBILIZATION IN PATIENTS WITH ANEURYSMAL SUBARACHNOID HAEMORRHAGE MAY IMPROVE FUNCTIONAL STATUS AND REDUCE CEREBRAL VASOSPASM RATE: A SYSTEMATIC REVIEW WITH META-ANALYSIS [file JRM-56-41225-s1.pdf]

Supplementary material has been published as submitted. It has not been copyedited, or typeset by Journal of Rehabilitation Medicine

Appendix S1 : MEDLINE search criteria

#	Searches
1	(early ambulation[MeSH Terms]) OR (walking[MeSH Terms]) OR (Occupational therapies [MeSH Terms]) OR ("neurological rehabilitation"[MeSH Terms]) OR (exercise therapy[MeSH Terms]) OR (ambulation, early[MeSH Terms])
2	("early ambulation"[Text Word]) OR (exercise therap*[Text Word]) OR ("neurological rehabilitation"[Text Word]) OR (ambulat*[Text Word]) OR ("early activit*"[Text Word]) OR ("early mobili*"[Text Word]) OR (verticaliz*[Text Word]) OR (physical therap*[Text Word]) OR (physiotherap*[Text Word]) OR ("early rehabilit*"[Text Word]) OR (walk*[Text Word]) OR ("occupational therap*"[Text Word])
3	(aneurysm, ruptured[MeSH Terms]) OR ("intracranial aneurysm"[MeSH Terms]) OR ("intracranial hemorrhages"[MeSH Terms])
4	("SAH"[Text Word]) OR (aSAH[Text Word]) OR (SAHs[Text Word])
5	(("haemorrhage"[Text Word]) OR ("hemorrhage"[Text Word]) OR (bleeding[Text Word])) AND (("subarachnoid"[Text Word]) OR (arachnoid[Text Word]) OR (intracranial[Text Word]))
6	("2001"[Date - Publication] : "2021"[Date - Publication])
7	("english"[Language]) OR (FRENCH[Language])
8	(#1 OR #2) AND (#3 OR #4 OR #5) AND (#6) AND (#7)

Appendix S2 : EMBASE search criteria

#	Searches
1	('subarachnoid hemorrhage'/de OR 'aneurysm rupture'/exp OR 'intracranial aneurysm'/exp OR 'brain hemorrhage'/de)
2	('mobilization'/exp OR 'physiotherapy'/exp OR 'kinesiotherapy'/exp OR 'neurorehabilitation'/exp OR 'occupational therapy'/exp OR 'walking'/exp OR 'early ambulat*':ti,ab,kw OR 'exercise therap*':ti,ab,kw OR 'neurological rehabilitation':ti,ab,kw OR 'occupational therap*':ti,ab,kw OR 'verticaliz*':ti,ab,kw OR 'ambulat*':ti,ab,kw OR 'early activit*':ti,ab,kw OR 'physical therap*':ti,ab,kw OR 'physiotherap*':ti,ab,kw OR 'early rehab*':ti,ab,kw OR 'walk*':ti,ab,kw OR 'early mobil*':ti,ab,kw)
3	1 and 2
4	([english]/lim OR [french]/lim) AND [2001-2021]/py
5	3 and 4
6	('case report'/de OR 'case study'/de)
7	5 not 6

Appendix S3 : PEDro search criteria

#	Searches
1	subarachnoid
2	intracranial bl*
3	SAH*
4	aSAH*
5	*arachnoid
6	ruptured aneurysm
7	cerebral aneurysm

Appendix S4 : CINAHL search criteria

#	Searches
1	MH ("Early Ambulation" OR "Occupational Therapy" OR "Physical Therapy" OR "Therapeutic Exercise+" OR "Walking")
2	TI ("early ambulation" OR "Physical Therap*" OR "neurological rehabilitation" OR "exercise therap*" OR "ambulat*" OR "early activit*" OR "early mobili*" OR "verticaliz*" OR "physiotherap*" OR "early rehabilitation" OR "walk*" OR "occupational therap*")
3	AB ("early ambulation" OR "Physical Therap*" OR "neurological rehabilitation" OR "exercise therap*" OR "ambulat*" OR "early activit*" OR "early mobili*" OR "verticaliz*" OR "physiotherap*" OR "early rehabilitation" OR "walk*" OR "occupational therap*")
4	SU ("early ambulation" OR "Physical Therap*" OR "neurological rehabilitation" OR "exercise therap*" OR "ambulat*" OR "early activit*" OR "early mobili*" OR "verticaliz*" OR "physiotherap*" OR "early rehabilitation" OR "walk*" OR "occupational therap*")
5	1 OR 2 OR 3 OR 4
6	MH ("Intracranial Hemorrhage+")
7	TI ("cerebral aneurysm" OR "ruptured aneurysm" OR "intracranial aneurysm" OR "SAH" OR "aSAH" OR "SAHs")
8	AB ("cerebral aneurysm" OR "ruptured aneurysm" OR "intracranial aneurysm" OR "SAH" OR "aSAH" OR "SAHs")
9	SU ("cerebral aneurysm" OR "ruptured aneurysm" OR "intracranial aneurysm" OR "SAH" OR "aSAH" OR "SAHs")
10	TI ("haemorrhag*" OR "hemorrhag*" OR "bleeding")
11	AB ("haemorrhag*" OR "hemorrhag*" OR "bleeding")

12	SU("haemorrhag*" OR "hemorrhag*" OR "bleeding")
13	TI ("subarachnoid" OR "arachnoid" OR "intracranial")
14	AB ("subarachnoid" OR "arachnoid" OR "intracranial")
15	SU ("subarachnoid" OR "arachnoid" OR "intracranial")
16	6 OR 7 OR 8 OR 9 OR 10 OR 11 OR 12 OR 13 OR 14 or 15
17	5 AND 16

Appendix S5 : CENTRAL search criteria

#	Searches
1	MeSH descriptor: [Intracranial Aneurysm]
2	MeSH descriptor: [Aneurysm, Ruptured]
3	MeSH descriptor: [Intracranial Hemorrhages]
4	#3 OR #2 OR #1
5	("SAH"):ti,ab,kw
6	("aSAH"):ti,ab,kw
7	("SAHs"):ti,ab,kw
8	("haemorrhage"):ti,ab,kw
9	(hemorrhage):ti,ab,kw
10	(bleeding):ti,ab,kw
11	(subarachnoid):ti,ab,kw
12	(arachnoid):ti,ab,kw
13	(intracranial):ti,ab,kw
14	#11 OR #12 OR #13
15	#8 OR #9 OR #10
16	#15 AND #14
17	#4 OR #5 OR #6 OR #7 OR #16

18	MeSH descriptor: [Early Ambulation]
19	MeSH descriptor: [Occupational Therapy]
20	MeSH descriptor: [Neurological Rehabilitation]
21	MeSH descriptor: [Exercise Therapy]
22	MeSH descriptor: [Walking]
23	("early ambulation"):ti,ab,kw
24	("neurological rehabilitation"):ti,ab,kw
25	(ambulat*):ti,ab,kw
26	(verticaliz*):ti,ab,kw
27	(early activit*):ti,ab,kw
28	(physical therap*):ti,ab,kw
29	(exercise therap*):ti,ab,kw
30	(physiotherap*):ti,ab,kw
31	(early rehabilitat*):ti,ab,kw
32	(walk*):ti,ab,kw
33	(occupational therap*):ti,ab,kw
34	#18 OR #19 OR #20 OR #21 OR #22 OR #23 OR #24 OR #25 OR #26 OR #27 OR#28 #29 OR #30 OR #31 OR #32 OR #33
35	#34 AND #17

Appendix S6 : WEB OF SCIENCE search criteria

#	Searches
1	TS= ("early ambulation" OR "exercise therap*" OR "neurological rehabilitation" OR "ambulat*" OR "early activit*" OR "early mobili*" OR "verticaliz*" OR "physical therap*" OR "physiotherap*" OR "early rehabilit*" OR "walk*" OR "occupational therap*")
2	TS= ("intracranial aneurysm" OR "ruptured aneurysm"OR "cerebral aneurysm" OR "SAH" OR "aSAH" OR "SAHs")
3	TS=("haemorrhag*" OR "hemorrhag*" OR "bleeding")
4	TS=("*arachnoid" OR "intracranial")
5	#3 AND #4
6	#2 OR #5
7	#1 AND #6
